# Single and mixed effects of seven heavy metals on stroke risk: 11,803 adults from National Health and Nutrition Examination Survey (NHANES)

**DOI:** 10.3389/fnut.2025.1524099

**Published:** 2025-03-12

**Authors:** Xinyi Huang, Yueran Wu, Yan Lu

**Affiliations:** ^1^Department of Epidemiology, School of Public Health, Nanjing Medical University, Nanjing, China; ^2^Jiujiang Center for Disease Control and Prevention, Jiujiang, China; ^3^Suzhou Centre for Disease Control and Prevention, Suzhou, China

**Keywords:** heavy metals, stroke, joint effect, National Health and Nutrition Examination Survey, exposure

## Abstract

**Background:**

The accumulation of heavy metals in soil and plants poses risks to food safety. Human exposure to heavy metals has been linked to stroke risk, though research on this connection is limited and findings are inconsistent.

**Methods:**

We estimated the associations of 7 blood metals [cadmium (Cd), lead (Pb), mercury (Hg), manganese (Mn), copper (Cu), selenium (Se), and zinc (Zn)] with the risk of stroke among 11,803 U.S. adults. Logistic regression account for the intricate sampling design and restricted cubic spline (RCS) was used to explore the associations between single heavy metal and stroke risk. The weighted quantile sum (WQS) and quantile g-computation (qgcomp) were employed to explore the joint effects of seven metals on stroke. Potential confounders were adjusted.

**Results:**

After adjusting for the potential confounders, the logistic regression analysis showed the log-transformed Cd and Zn level was associated with stroke (All *p* < 0.05). After adjusting for the potential confounders, the logistic regression analysis showed the log-transformed Cd and Zn level was associated with stroke (All *p* < 0.05). WQS and qgcomp analyses consistently demonstrated a positive correlation between metals-mixed exposure and stroke risk, identifying Cd and Cu as key contributors to the outcomes, while Zn may serve as a protective factor.

**Conclusion:**

These findings indicated that heavy metal exposure is associated with stroke risk, and the protective effect of Zn on stroke risk deserves further research to verify.

## 1 Introduction

Stroke is closely associated with the DALYs (disability-adjusted life-years) lost. And it is also the leading cause of death and disease burden worldwide. In the European Union, it is estimated that the number of people with stroke increased by 27% between 2017 and 2047 (1). Although the data about declining stroke incidence were encouraging, ageing population, accumulating risk factors, and improved survival rates led to an increased lifetime risk of stroke.

The etiology and pathogenesis of stroke are complex and diverse. There are multiple mechanisms involved, such as inflammation, oxidative stress, and ionic imbalance (2, 3). Exposure to metals, such as cadmium (Cd), lead (Pb), mercury (Hg), manganese (Mn), copper (Cu), selenium (Se), and zinc (Zn), may be associated with increased stroke incidence (4–11). Air inhalation, water and food intake, and dermal contact are the main exposure routes to environmental metals for the general population (12). Some toxic metals, such as arsenic and cadmium, may cause organs damage by oxidative stress, cell dysfunction, inflammation (13–15). Some essential elements, such as iron and selenium, have an indispensable role in human physiology and metabolism, but their excess presence may also result in various adverse effects (16).

Despite increasing interest, significant gaps remain in understanding the contributions of heavy metals to stroke risk. Current research primarily focuses on individual metals, leaving their combined effects largely unexplored. The impact of metals like cadmium on stroke remains inconclusive due to complex interactions between multiple metals. Given that heavy metals coexist in the environment with additive, synergistic, or antagonistic effects, more comprehensive studies are needed to assess their combined impact on stroke risk.

Therefore, this study was conducted to examine the individual and joint effects of seven heavy metals (Cd, Pb, Hg, Mn, Cu, Se, and Zn) on stroke comprehensively based on the data from the National Health and Nutrition Examination Survey (NHANES) 2011–2016. This research aims to evaluate the relationship between multiple heavy metals and stroke risk, focusing on their potential joint effects. The findings could provide insights into stroke prevention and risk assessment, guiding more effective public health strategies for environmental heavy metal exposure.

## 2 Methods

### 2.1 Study design and population

The NHANES is a national, biennial cross-sectional survey to assess the health and nutritional status of the general populations in the U.S., using a multistage, probability-sampling design to generate population-level estimates. It examines a nationally representative sample of 5,000 persons selected from 15 different locations. All study protocols and results are available on the website of the American Centers for Disease Control and Prevention (CDC).^1^ The survey was approved by the NCHS Research Ethics Review Board and all participants provided informed consent.

Data from three cycles (2011–2012, 2013–2014, and 2015–2016) of NHANES were combined in the study. Participants who were not tested for heavy metals were excluded. Dependent variable was the self-reported history of stroke. Participants with missing information on self-reported stroke outcomes were also excluded. Finally, 11,803 participants aged ≥20 years were involved in the final analysis.

### 2.2 Measurement of seven heavy metals

Pb, Cd, Hg, and Mn content of whole blood specimens were measured using mass spectrometry after a simple dilution sample preparation step. Inductively coupled plasma dynamic reaction cell mass spectrometry (ICP-DRC-MS) was used to measure the entire panel of the serum concentrations of Cu, Se, and Zn. According to the NHANES standard, the limit of detection (LOD) divided by the square root of two was used to replace the values below the LOD.

### 2.3 Covariates

Covariates included demographics such as age, gender (male or female), race (Mexican American, Non-Hispanic White, Non-Hispanic Black, Non-Hispanic Asian, Other Hispanic), family income to poverty ratio(a measure of family income relative to poverty guidelines specific to the survey year), body mass index (kg/m^2^), smoking status (≥100 or < 100 cigarettes/entire life), alcohol consumption (<12 or ≥ 12 drinks/year), and disease histories (yes or no) of coronary heart disease and diabetes. Data of covariates were collected through home interview, laboratory measurements, and questionnaires.

### 2.4 Statistical analysis

According to the NHANES standard, we used the 2011–2016 sample weights of NHANES to calculate the new weights of the study samples. The PROC SURVEY procedure was employed to account for the intricate sampling design of NHANES, which included weight, cluster, and strata statements, to incorporate sampling weights. Data on seven metals were natural log 10 transformed to normalize their distribution for analysis. Statistical analyses were performed with SAS software (V.9.4).

To explore the non-linear relationship, restricted cubic spline (RCS) with three knots coupled with a logistic regression model was to assess the dose–response relationship between single and multiple metals (continuous variables) and stroke. In addition, the RLM function was used to test the nonlinear effect of the model. The R package (“rms”) was used to achieve this.

WQS regression was adopted to estimate the association between multiple-metal exposure and stroke (17). We used the R package (“gWQS”) to calculate the WQS index comprised of weighted sums of individual metal concentrations. Heavy metals are often correlated with one another, and traditional regression models can struggle with multicollinearity, leading to unreliable estimates. It is important to note that all heavy metals in the model are assumed to have the same direction of association with the outcome. Qgcomp, an adaptive modeling method with weighted quantile sum (WQS) regression, unlike the WQS model, does not assume a fixed direction and can evaluate the differing directions of mixed effects for individual exposures (18). To investigate the difference of heavy metal mixing effect between gender, age and BMI, stratified analysis was used (BMI was divided into two groups by 24 kg/m^2^, and age was divided into two groups by 60 years). The R package (“qgcomp”) was used to achieve this.

All models were adjusted for gender, age, race/ethnicity, family income-to-poverty ratio, drinking alcohol status, smoking status, body mass index, diabetes, and coronary heart disease. Missing covariate values were processed using multiple imputation. *p* value <0.05 was considered significant.

## 3 Results

### 3.1 Characteristics of participants

A total of 11,803 participants (438 were diagnosed with stroke) with complete data on heavy metals and stroke were included in this study. The survey-weighted descriptive statistics are presented in Table 1. The weighted mean age of participants was 49.14 years, and 48.45% (*n* = 5,781) were male. Overall, age, ethnicity, drinking alcohol status, smoking status, family PIR, and comorbidities (diabetes and coronary heart disease) were statistically significant differences between stroke and non-stroke participants (*p* < 0.05). Stroke participants had higher proportion of diabetes, coronary heart disease prevalence, older age, and smoking status with ≥100 cigarettes/entire life. The non-stroke participants had higher proportion of drinking alcohol status with ≥12 drinks/year, and higher family PIR. The participants’ weighted mean concentrations of Cd, Pb, Hg, Mn, Cu, Se, and Zn was 0.34 ± 0.49 μg/L, 1.19 ± 1.499 μg/L, 1.17 ± 2.169 μg/L, 10.38 ± 3.919 μg/L, 118.85 ± 29.189 μg/L, 187.32 ± 26.419 μg/L, and 81.80 ± 15.229 μg/L, respectively. The detection rate of all metals was greater than 75%.

**Table 1:** Basic characteristics of participants (aged ≥20) by stroke from NHANES 2011–2016.

Characteristics	Total	Stroke	Non-stroke	*p* value
*N*		438	11,365	
Age (years, mean ± SE)	49.14 ± 17.75	66.42 ± 12.68	48.48 ± 17.59	<0.001
Gender, *n* (%)				0.94
Male	5,718 (48.45)	213 (48.63)	5,505 (48.44)	
Female	6,085 (51.55)	225 (51.37)	5,860 (51.56)	
Race/ethnicity, *n* (%)				<0.001
Mexican American	1,516 (12.84)	39 (8.90)	1,477 (13.00)	
Other Hispanic	1,276 (10.81)	32 (7.31)	1,244 (10.95)	
Non-Hispanic White	4,430 (37.53)	200 (45.66)	4,230 (37.22)	
Non-Hispanic Black	2,714 (22.99)	127 (29.00)	2,587 (22.76)	
Non-Hispanic Asian	1867 (15.82)	40 (9.13)	1827 (16.08)	
Drinking alcohol status, (drinks/year), n (%)				0.003
≥12	7,258 (71.36)	243 (64.63)	7,015 (71.62)	
<12	2,913 (28.64)	133 (35.37)	2,780 (28.38)	
Smoking status, (cigarettes/entire life), *n* (%)				<0.001
≥100	5,081 (43.09)	262 (59.82)	4,819 (42.44)	
<100	6,711 (56.91)	176 (40.18)	6,535 (57.56)	
Family PIR (mean ± SE)	2.44 ± 1.65	1.93 ± 1.36	2.46 ± 1.66	<0.001
BMI (kg/m^2^, mean ± SE)	29.16 ± 7.08	29.33 ± 6.82	29.15 ± 7.09	0.63
Coronary heart disease, *n* (%)				<0.001
Yes	461 (3.92)	83 (19.35)	378 (3.34)	
No	11,301 (96.08)	346 (80.65)	10,955 (96.66)	
Diabetes, *n* (%)				<0.001
Yes	1,608 (13.96)	144 (34.45)	1,464 (13.19)	
No	9,909 (86.04)	274 (65.55)	9,635 (86.81)	
Cd (μg/L, mean ± SE)	0.34 ± 0.49	0.39 ± 0.50	0.34 ± 0.49	0.06
Pb (μg/dL, mean ± SE)	1.19 ± 1.49	1.20 ± 1.51	1.19 ± 1.49	0.94
Hg (μg/L, mean ± SE)	1.17 ± 2.16	1.19 ± 1.97	1.17 ± 2.17	0.81
Mn (μg/L, mean ± SE)	10.38 ± 3.91	10.51 ± 4.83	10.38 ± 3.87	0.59
Cu (μg/dL, mean ± SE)	118.85 ± 29.18	121.80 ± 27.32	118.70 ± 29.25	0.18
Se (μg/L, mean ± SE)	187.32 ± 26.41	187.70 ± 24.49	187.30 ± 26.48	0.74
Zn (μg/dL, mean ± SE)	81.80 ± 15.22	79.37 ± 15.64	81.91 ± 15.20	0.035

Family PIR, family income to poverty ratio; BMI, body mass index; Pb, lead; Cd, cadmium; Hg, total mercury; Mn, manganese; Se, selenium; Cu, copper; Se, selenium; Zn, zinc.

### 3.2 Multivariable logistic model

The individual effects of seven metals on stroke using the survey-weighted logistic regression are provided in Table 2. After adjusting for the potential confounders, the log-transformed Cd and Zn level was associated with stroke (Cd: OR = 1.13, 95% CI: 1.01, 1.26; Zn: OR = 0.17, 95% CI: 0.05, 0.58). The highest exposure quantile of blood Cd increased the risk of stroke compared to quantile 1 (OR = 1.54, 95% CI: 1.02, 2.30; *P* trend = 0.004). While highest exposure quantile of blood Zn decreased the risk of stroke compared to quantile 1 (OR = 0.47, 95% CI: 0.24, 0.92; *P* trend = 0.024). Besides, there was an elevated stroke risk in Q3 of Cd (OR = 1.88, 95% CI: 1.29, 2.73), Q2 of Hg (OR = 1.98, 95% CI: 1.17, 3.34), compared to Q1. And there was a lower stroke risk in Q2 of Mn (OR = 0.68, 95% CI: 0.47, 0.96), compared to Q1.

**Table 2:** Associations of single blood metals with stroke risk, NHANES 2011–2016.

Metals	Stroke/non stroke	Continuous		Q1	Q2		Q3		Q4		*P* for trend
	438/11,365	Unadjusted OR (95% CI)	Adjusted OR (95% CI)	ref	Unadjusted OR (95% CI)	Adjusted OR (95% CI)	Unadjusted OR (95% CI)	Adjusted OR (95% CI)	Unadjusted OR (95% CI)	Adjusted OR (95% CI)	
Cd	387/9921	**1.14 (1.02,1.26)**	**1.13 (1.01,1.26)**	–	1.26 (0.80,1.98)	1.31 (0.83,2.08)	**1.84 (1.29,2.63)**	**1.88 (1.29,2.73)**	**1.55 (1.04, 2.30)**	**1.54 (1.02, 2.30)**	**0.004**
Pb	387/9921	1.01 (0.86,1.19)	0.99 (0.84,1.17)	–	0.94 (0.64,1.37)	0.98 (0.65,1.48)	1.23 (0.79,1.93)	1.24 (0.77,2.00)	1.02 (0.71,1.46)	0.99 (0.69,1.42)	0.707
Hg	387/9921	1.08 (0.94,1.23)	1.08 (0.94,1.24)	–	**1.99 (1.25,3.16)**	**1.98 (1.17, 3.34)**	1.61 (0.99,2.61)	1.61 (0.95, 2.73)	1.55 (0.98,2.47)	1.52 (0.94, 2.46)	0.260
Mn	386/9921	0.96 (0.62,1.48)	1.02 (0.64,1.62)	–	**0.67 (0.47, 0.96)**	**0.68 (0.47, 0.96)**	0.75 (0.49,1.14)	0.72 (0.47, 1.11)	0.87 (0.60,1.26)	0.94 (0.63, 1.41)	0.843
Cu	167/3987	1.45 (0.72,2.90)	1.72 (0.77,3.86)	–	0.94 (0.47,1.88)	0.95 (0.44,2.04)	1.29 (0.71,2.34)	1.44 (0.76, 2.72)	1.20 (0.65, 2.20)	1.29 (0.66, 2.55)	0.268
Se	386/9921	0.81 (0.27,2.45)	0.70 (0.23,2.18)	–	0.96 (0.65,1.42)	0.88 (0.57,1.36)	0.95 (0.70,1.28)	0.88 (0.64, 1.21)	1.09 (0.74, 1.59)	1.02 (0.68,1.54)	0.913
Zn	167/3987	**0.22 (0.07,0.73)**	**0.17 (0.05,0.58)**	–	0.75 (0.35, 1.60)	0.70 (0.32,1.53)	0.62 (0.35,1.11)	0.60 (0.32, 1.11)	**0.51 (0.27, 0.98)**	**0.47 (0.24, 0.92)**	**0.024**

Models were adjusted for gender, age, race/ethnicity, family income-to-poverty ratio, drinking alcohol status, smoking status, body mass index, diabetes, and coronary heart disease. Continuous, Ln-transformed concentration of metal; Stroke/non-stroke, the ratio of people with and without Stroke; Q, quartile; Pb, lead; Cd, cadmium; Hg, mercury; Mn, manganese; Se, selenium; Cu, copper; Zn, zinc. The bold values represent *P*<0.05.

### 3.3 Nonlinear relationship between metals and stroke

**Figure 1:**
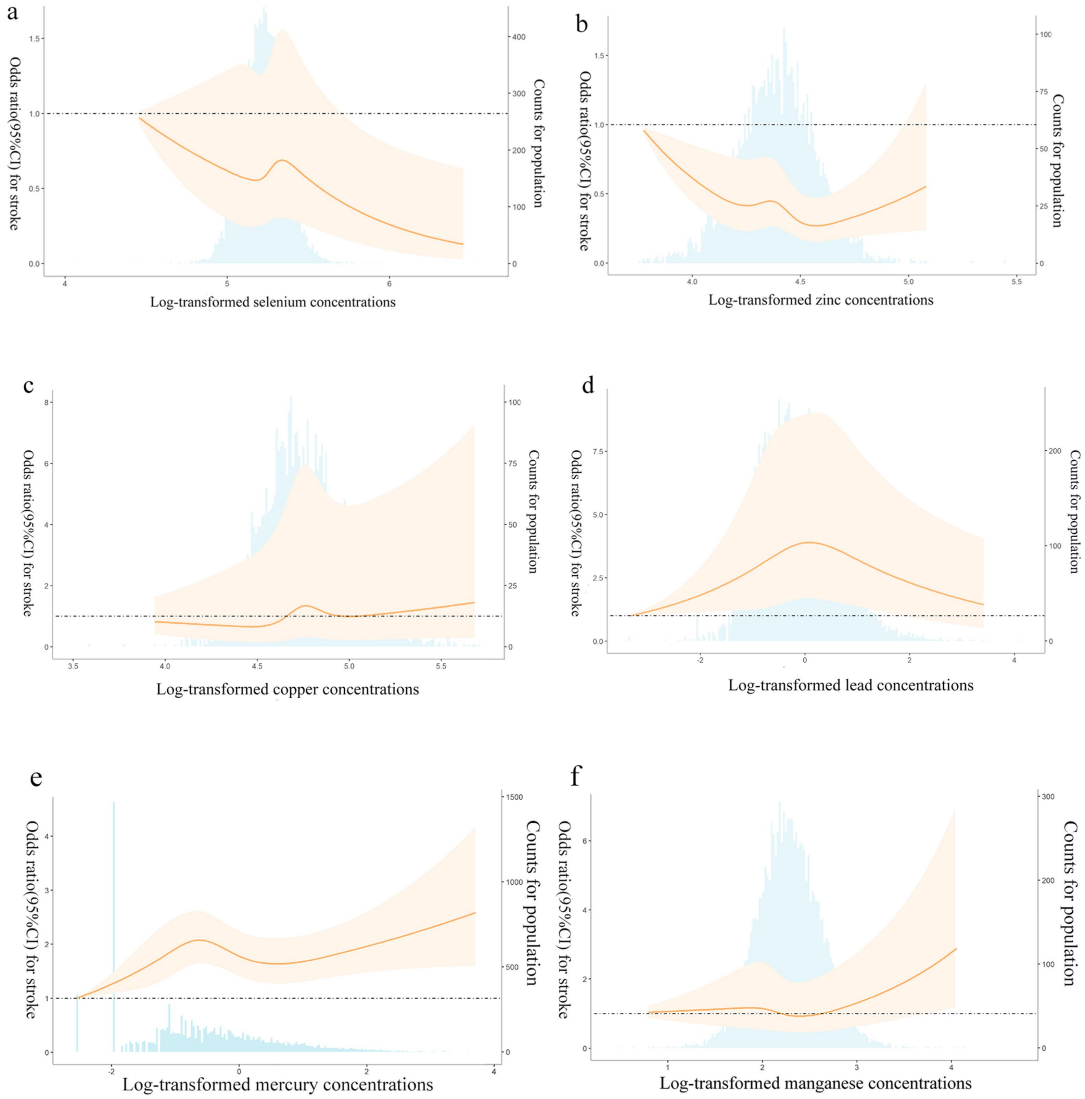
**(a–f)** Adjusted restricted cubic spline (RCS) for the association between metals and stroke. The lines with shaded represent adjusted hazard ratios [95% confidence interval (CI)] based on RCS for the log-transformed levels of selenium **(a)**, zinc **(b)**, copper **(c)**, lead **(d)**, mercury **(e)**, manganese **(f)** in the model, with the reference value was set at the minimum. Adjustment factors were gender, age, race/ethnicity, family income-to-poverty ratio, drinking alcohol status, smoking status, body mass index, diabetes, and coronary heart disease. The bars represent histograms of metal distribution among the participants.

Figures 1a–f showed the results of the restricted cubic spline analyses. The results showed that except Cd, the other six heavy metals had nonlinear association with stroke risk (*P* Zn for nonlinearity = 0.004, *P* Se for nonlinearity = 0.007, *P* Cu, Mn, Hg and Pb for nonlinearity<0.001). Stroke risk decreased with increasing Se, while increasing Cu, Hg, and Mn can increase stroke risk. We also found a suggestion of U-shaped associations between Zn concentrations and stroke risk.

### 3.4 WQS regression analysis

**Figure 2:**
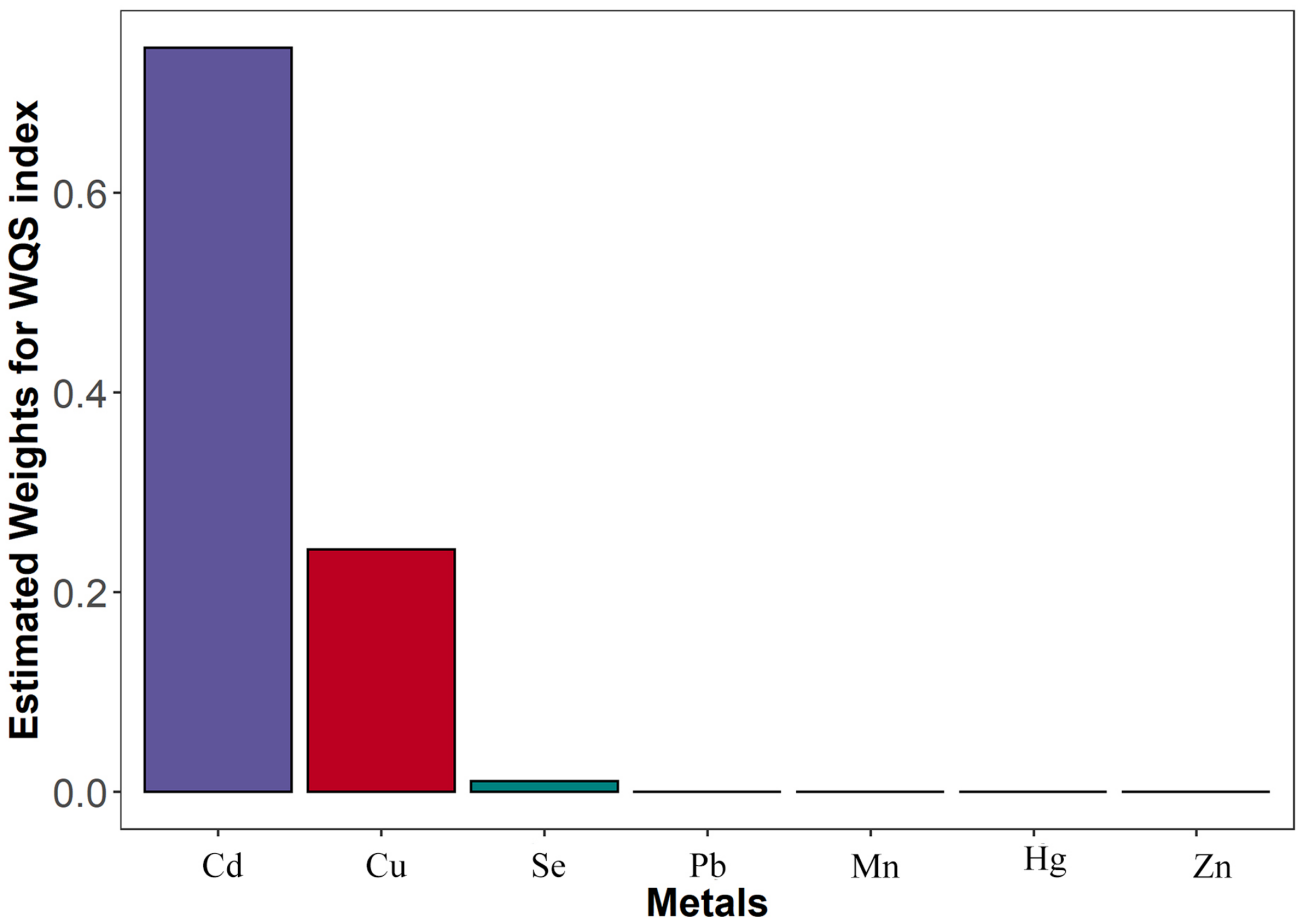
The WSQ regression estimated weights of each of the seven metals associated with stroke risk. Models were adjusted for gender, age, race/ethnicity, family income-to-poverty ratio, drinking alcohol status, smoking status, body mass index, diabetes, and coronary heart disease. WQS, weighted quantile sum; Pb, lead; Cd, cadmium; Hg, total mercury; Mn, manganese; Se, selenium; Cu, copper; Zn, zinc.

WQS regression analysis showed associations between all seven metals (Pb, Cd, Hg, Mn, Cu, Se, and Zn) co-exposure and stroke (*p* < 0.001). According to the logistic results, the preset beta values was positive. The estimated weights for the seven metals are provided in Figure 2. Blood Cd was the highest weighted metal in general population, followed by blood Cu.

### 3.5 Quantile-based g-computation analysis

**Figure 3:**
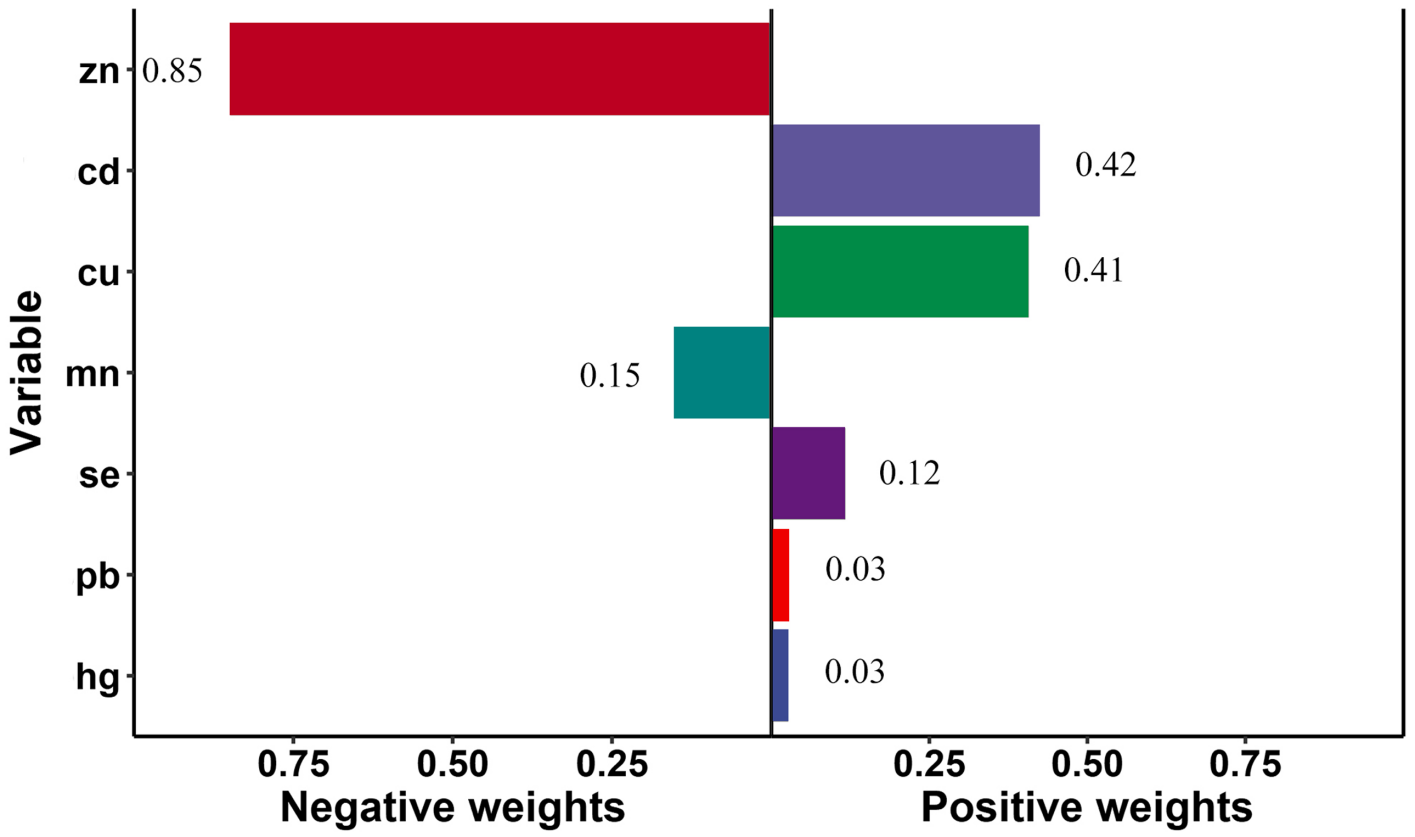
Quantile g-computation regression analysis of the relationship between seven metals associated and stroke risk among all participants. Models were adjusted for gender, age, race/ethnicity, family income-to-poverty ratio, drinking alcohol status, smoking status, body mass index, diabetes, and coronary heart disease.

Qgcomp analyzed the estimated exposure weights in the positive and negative directions, respectively. The joint weighted value of exposure was significantly associated with stroke risk (OR = 1.10, 95%CI: 1.01, 1.21; *p* < 0.05). The metal of largest positive weight in stroke risk was Cd (42.4%), followed by Cu (40.6%), while Zn (84.8%) was the largest negative weight metal in stroke risk (Figure 3). And there was a significant correlation between metals and stroke risk in smoke group (OR = 1.38, 95%CI: 1.22, 1.55; *p* < 0.05), young group (OR = 1.46, 95%CI: 1.23, 1.73; *p* < 0.05), and female group (OR = 1.17, 95%CI: 1.03, 1.33; *p* < 0.05).

**Figure 4:**
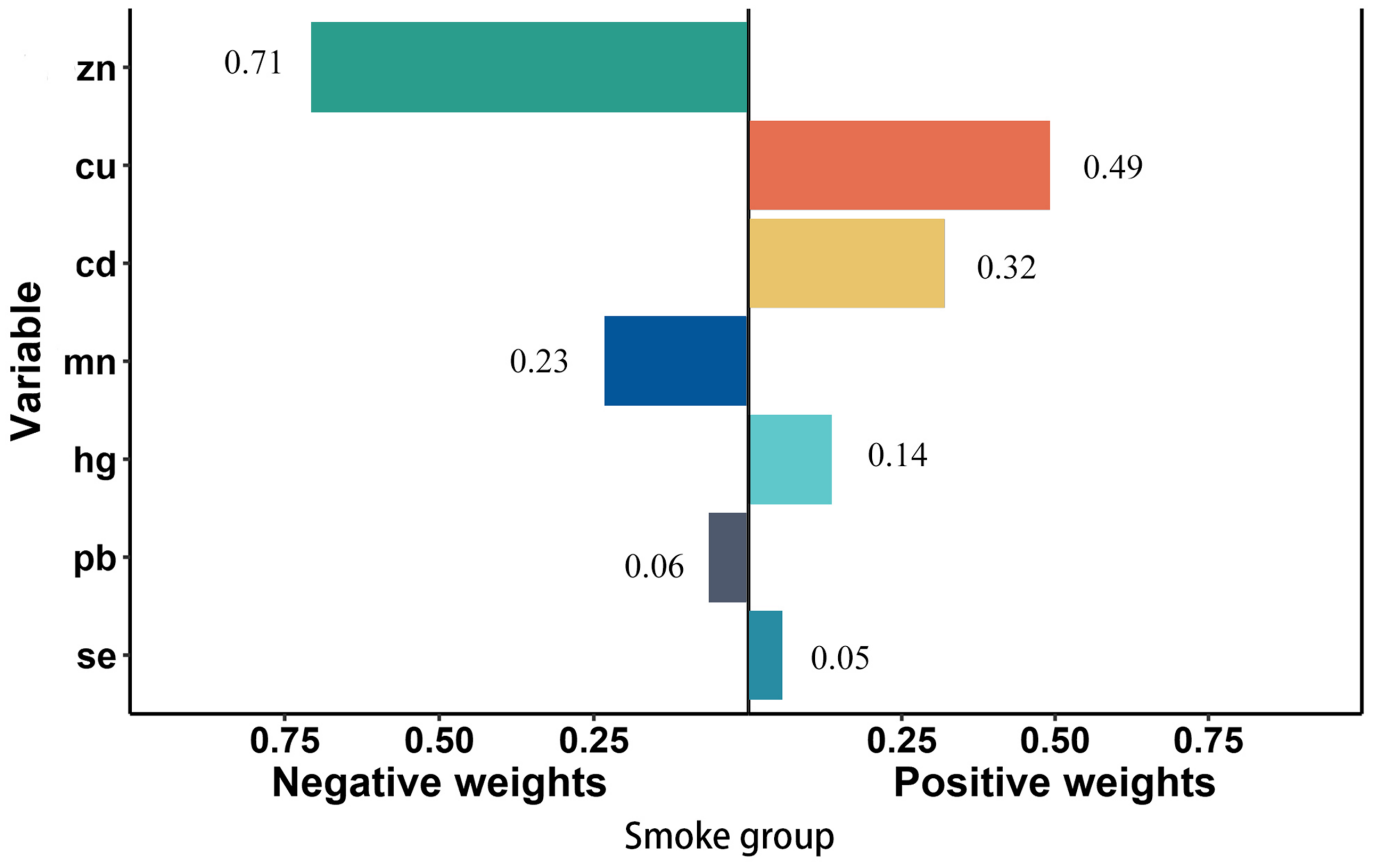
Quantile g-computation regression analysis of the relationship between seven metals associated and stroke risk among smoke participants. Models were adjusted for gender, age, race/ethnicity, family income-to-poverty ratio, drinking alcohol status, body mass index, diabetes, and coronary heart disease.

**Figure 5:**
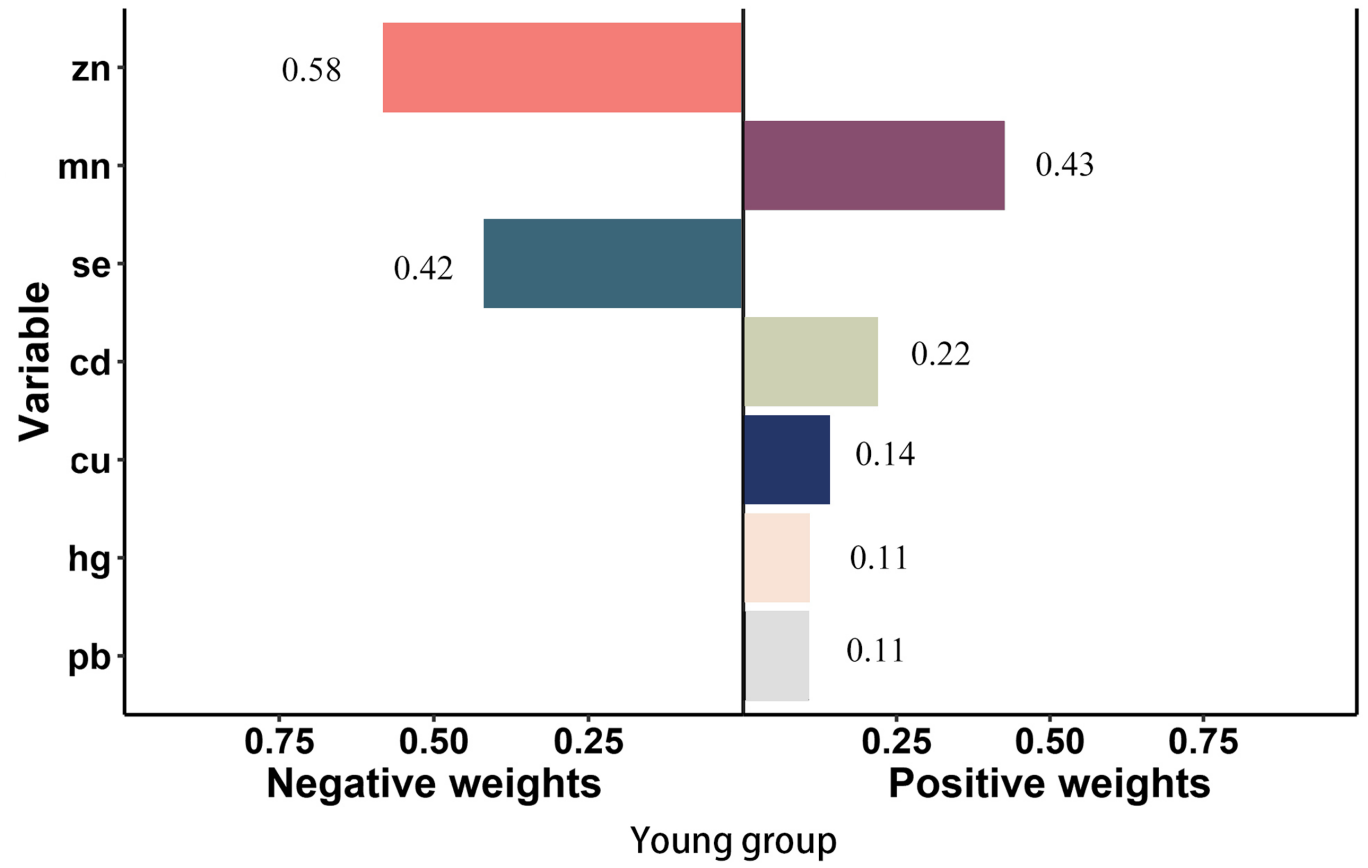
Quantile g-computation regression analysis of the relationship between seven metals with stroke risk among young. Models were adjusted for gender, age, race/ethnicity, family income-to-poverty ratio, drinking alcohol status, smoking status, body mass index, diabetes, and coronary heart disease. In subgroup analyses among female and male, gender was not adjusted. In subgroup analyses among young and elderly people, age was not adjusted.

**Figure 6:**
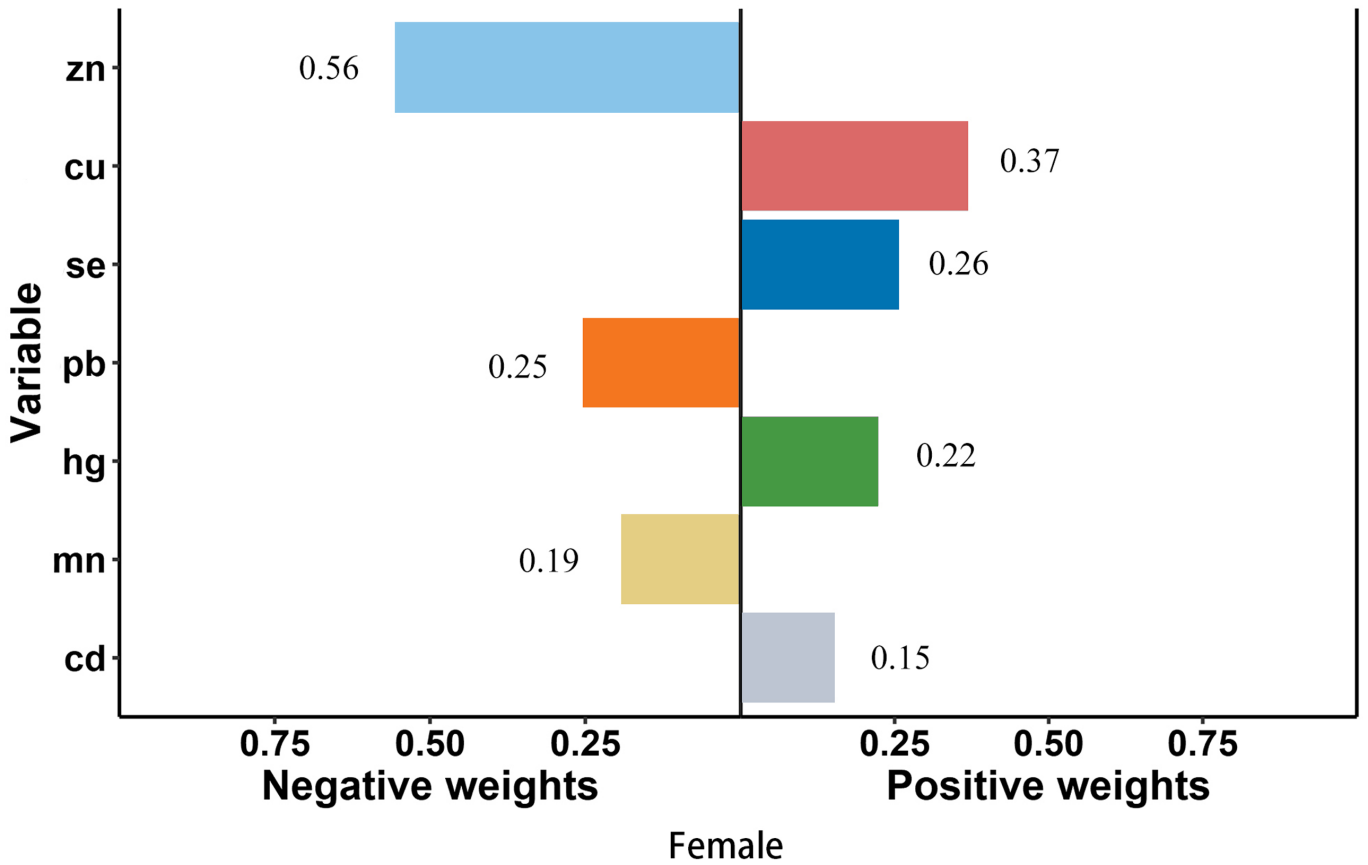
Quantile g-computation regression analysis of the relationship between seven metals with stroke risk among female. Models were adjusted for gender, age, race/ethnicity, family income-to-poverty ratio, drinking alcohol status, smoking status, body mass index, diabetes, and coronary heart disease. In subgroup analyses among female and male, gender was not adjusted. In subgroup analyses among young and elderly people, age was not adjusted.

In smoke group, Cu (weighted 49.1%) was the most positive weighted metals contributing to the stroke risk, followed by Cd (weighted 32.0%). Zn (weighted 70.5%) was the most negative weighted metals (Figure 4). In young group, Mn and Cd (weighted 42.6 and 21.9%) were the most positive weighted metals contributing to the stroke risk, while Zn (weighted 58.1%) was the most negative weighted metals, followed by Se (weighted 41.9%) (Figure 5). In female group, Zn (weighted 55.5%) was also the most negative weighted metals contributing to the stroke risk, the metal of largest positive weight in stroke risk was Cu (weighted 36.9%) (Figure 6). The results of other subgroups were not statistically significant (Supplementary Figures S1–S5).

## 4 Discussion

The accumulation of heavy metals in soil and plant tissues is a significant concern, as it affects both soil quality and food safety, potentially impacting human health through the food chain. This study employed various statistical methods to assess the complex relationships between metals (Cd, Pb, Hg, Mn, Cu, Se, and Zn) and stroke risk, highlighting the association between mixed metal exposure and stroke risk.

After adjusting for potential confounders, logistic regression analysis showed that Cd was positively associated with stroke risk, while Zn was negatively associated with stroke risk. Restricted cubic spline analysis showed that a suggestion of U-shaped associations between Zn concentrations and stroke risk, which may indicate there is an optimal concentration of Zn for the protective effect of stroke.

As components of several metalloenzymes, Zn is also an essential trace element (19). The prevention of free radicals has been the focus of numerous studies (20–22). Zn has antioxidant and anti-inflammatory properties in the brain (23, 24), protecting cells from free radical damage. Besides, the essential role of Zn in endothelial integrity has been studied (25). Its deficiency leads to severe damage to the endothelial protective function, causing or enhancing a cytokine-mediated inflammatory process. Zinc also plays a critical role in promoting the formation of atherosclerotic plaque (26). In addition, its excessive release and accumulation in microvessels also leads to ischemia-induced neuronal and vascular injury (27). Abnormal accumulation of Zn in the brain has been found in various neurological diseases, including stroke (28). Although many in vivo experiments have confirmed the protective effect of Zn on stroke, epidemiological evidence remains controversial, and the impact of zinc on different stroke subtypes is still unclear (29–31).

Further, multiple methods were applied to evaluate the effect of metals co-exposure on stroke risk. The findings of WQS regression suggested evident associations between co-exposure to all the seven metals and stroke risk, which were mainly driven by Cd and Cu.

It is estimated that 5 million people are exposed chronically to Cd. Cd from mining, smelting, and industrial waste can also pollute air, water, and soil, leading to the contamination of foods such as rice, wheat, potatoes, leafy vegetables, fish, and shellfish. Remarkably, tobacco smoking can further increase Cd exposure (32–35). Cd exposure is thought to cause stroke through multiple mechanisms. Cd toxicity is achieved by consumption of glutathione and binding to protein sulfhydryl groups (36, 37). Cd has also been demonstrated to impair endothelial function (38, 39). The study have shown that Cd exposure may be an independent risk factor for ischemic stroke in the US general population. And the potential adverse effects of Cd exposure can be ameliorated by never smoking and high serum zinc levels (40). In addition, Cd exposure is associated with increased morbidity and mortality in stroke (41, 42). The existence of a relationship between high blood Cd levels and prevalent stroke was found in the Korean population under age 60 (43). In our study, the metal of largest positive weight in stroke risk was Cd (weighted 42.4%) among general population.

Cu is an essential element in the redox metabolism. Its deficiency increases oxidative stress and might be associated with oxidative damage to DNA, fats, and proteins (44, 45). In addition, Cu is critical trace elements in the impairment of endothelial function (46–48) and processes of inflammation (49), which may involve in carotid plaques and further lead to cerebral ischemic injury. A meta-analysis has indicated that serum Cu may be a risk factor of ischemic stroke (50). Numerous studies have demonstrated a positive correlation between serum Cu levels and the risk of ischemic stroke (11, 51). In patients with hypertension, baseline plasma Cu was significantly associated with the risk of first stroke, especially in people with higher BMI (52).

However, there are differences in results across subgroups. For example, in the young group, Mn (weighted 42.6%) was the metal with the most positive weight contributing to stroke risk, followed by Cd (weighted 21.9%). Mn, a vital component in many enzyme-driven metabolic functions, acts as a catalyst for various enzymatic reactions, including polysaccharide polymerase, liver arginase, cholinesterase, and pyruvate carboxylase (53, 54). Currently, little attention has been devoted to the relation between Mn and stroke. The research suggested that the association between plasma Mn concentration and ischemic stroke was significant in the single-metal model, however, the association was insignificant in the multiple-metal model (55). The differences in metal contributions to stroke risk between the general population and the young group may be attributed to variations in exposure sources, biological responses, and lifestyle factors. Cd may have a larger contribution in the general population due to more widespread exposure, while Mn may have a greater impact in the young group due to differences in exposure patterns and susceptibility.

## 5 Conclusion

In conclusion, heavy metals play a significant role in stroke pathogenesis. The study identifies a positive correlation between mixed metal exposure, particularly Cd and Cu, and stroke risk, while Zn may offer protective effects. Future research should focus on the toxic effects of Cd and Cu, and the optimal dosages and mechanisms through which Zn provides protection. Understanding these will help develop interventions to reduce stroke risk.

## Data Availability

The datasets presented in this study can be found in online repositories. The names of the repository/repositories and accession number(s) can be found at: http://www.cdc.gov/nchs/nhanes.htm.

## Footnotes

^1^ http://www.cdc.gov/nchs/nhanes.htm
