# Supplementary material for: Single and mixed effects of seven heavy metals on stroke risk: 11,803 adults from National Health and Nutrition Examination Survey (NHANES)
Source: Front Nutr. 2025 Mar 12;12:1524099. doi: 10.3389/fnut.2025.1524099 (PMC11937853; doi:10.3389/fnut.2025.1524099)
Supplement: Supplementary file 1 [file Image_1.pdf]

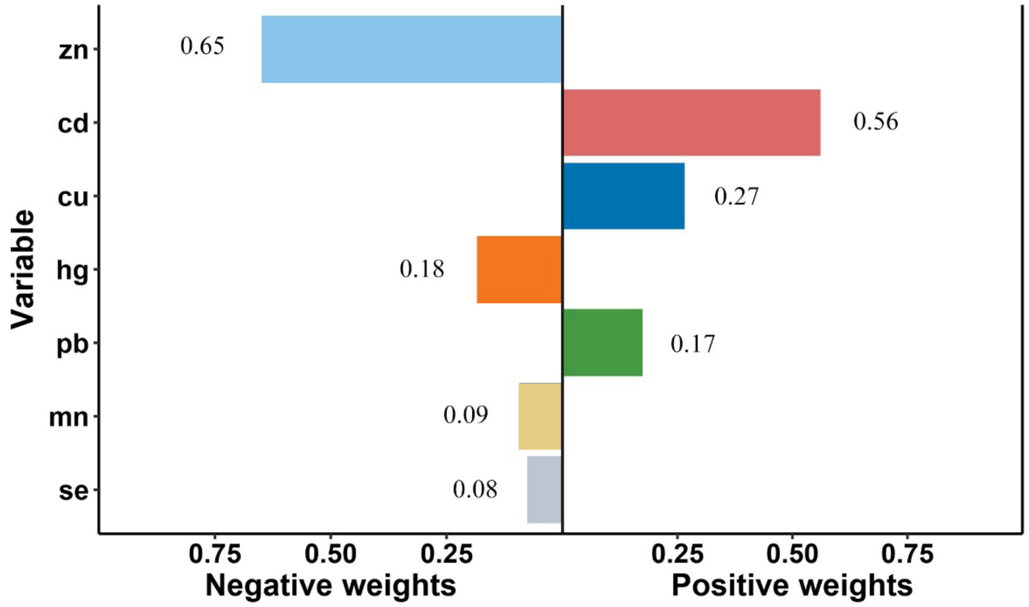

Figure S1. Quantile g-computation regression analysis of the relationship between bisphenol levels and the risk of preterm birth among male. Models were adjusted for age, race/ethnicity, family income-to-poverty ratio, drinking alcohol status, smoking status, body mass index, diabetes, and coronary heart disease. OR= 1.06; 95%CI (0.93,1.20); P=0.39.

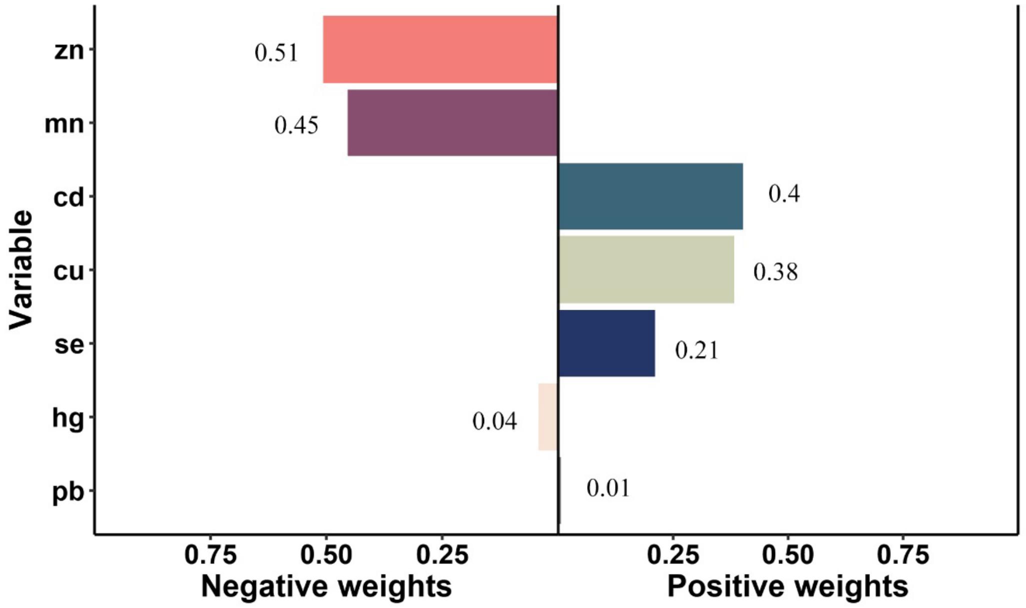

Figure S2. Quantile g-computation regression analysis of the relationship between bisphenol levels and the risk of preterm birth among elder. Models were adjusted for sex, race/ethnicity,

family income-to-poverty ratio, drinking alcohol status, smoking status, body mass index, diabetes, and coronary heart disease. OR= 1.10; 95%CI (0.99,1.23); P=0.09.

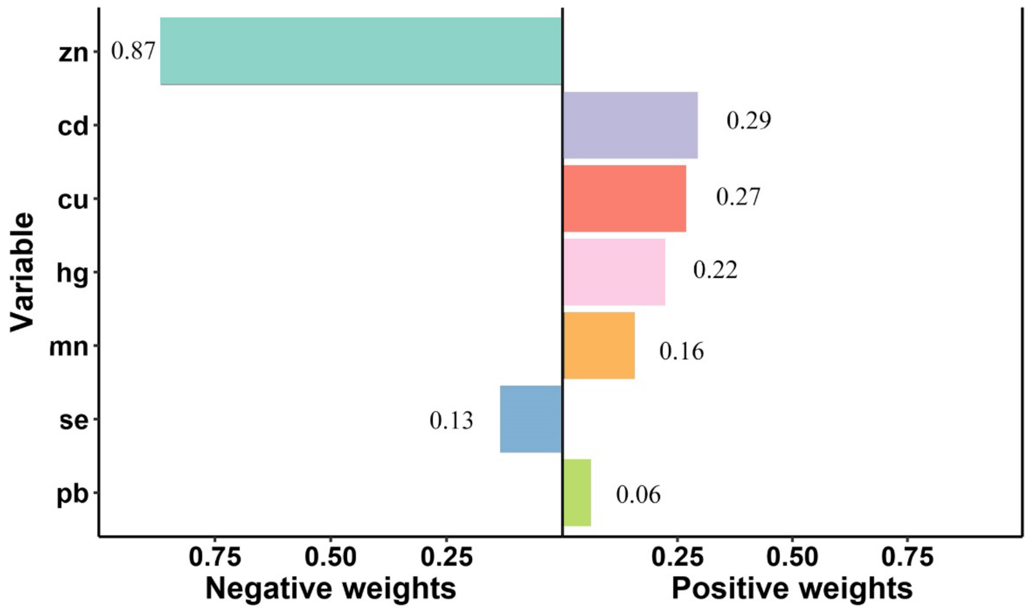

Figure S3. Quantile g-computation regression analysis of the relationship between bisphenol levels and the risk of preterm birth among participants with low BMI. Models were adjusted for sex, age, race/ethnicity, family income-to-poverty ratio, drinking alcohol status, smoking status, diabetes, and coronary heart disease. OR= 0.97; 95%CI (0.82,1.15); P=0.70.

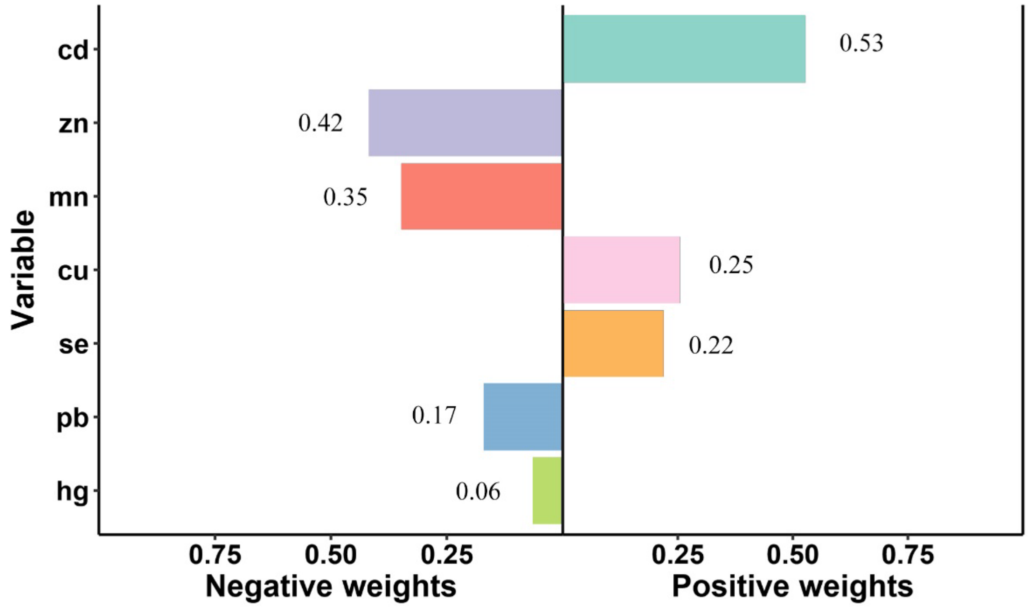

Figure S4. Quantile g-computation regression analysis of the relationship between bisphenol levels and the risk of preterm birth among participants with high BMI. Models were adjusted for sex, age, race/ethnicity, family income-to-poverty ratio, drinking alcohol status, smoking status, diabetes, and coronary heart disease. OR= 1.08; 95%CI (0.97,1.20); P=0.14.

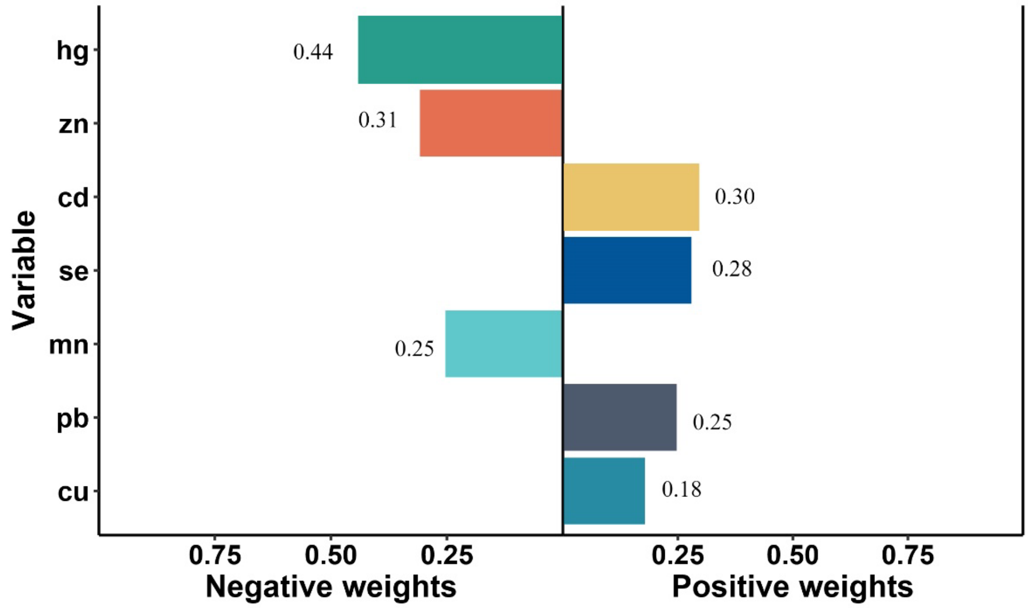

Figure S5. Quantile g-computation regression analysis of the relationship between bisphenol levels and the risk of preterm birth among participants with non-smoke. Models were adjusted for sex, age, race/ethnicity, family income-to-poverty ratio, drinking alcohol status, body mass index, diabetes, and coronary heart disease. OR= 1.07; 95%CI (0.92,1.23); P=0.38.
